# Heterotropic Effect of *β*-lactam Antibiotics on Antioxidant Property of Haptoglobin (2-2)-Hemoglobin Complex

**Published:** 2011

**Authors:** Masoumeh Tayari, Zahra Moosavi-Nejad, Fatemeh Moosavi Nejad, Mostafa Rezaei-Tavirani, Marzieh Dehghan Shasaltaneh

**Affiliations:** a *Proteomics Research Center, Shahid Beheshti University of Medical Sciences, Tehran, Iran.*; b * Department of Biology, Faculty of Basic Sciences, Alzahra University, 1993891176, Tehran, Iran.*; c * Department of Anatomy, Faculty of Medicine, Fukuoka University, 7-45-1 Nanakuma, Jonanku, Fukuoka 814-0180, Japan.*; d * Asre Novin Institute of Research and Industrial Services, Tehran, Iran.*

**Keywords:** Haptoglobin, Hemoglobin, Antioxidant, Antibiotics, Beta-lactam, Non-Michaelis

## Abstract

Haptoglobin (Hp) is a mammalian serum glycoprotein showing a genetic polymorphism with three types, 1-1, 2-2 and 1-2. Hp appears to conserve the recycling of heme-iron by forming an essentially irreversible but non-covalent complex with hemoglobin which is released into the plasma by erythrocyte lysis. As an important consequence, Haptoglobin-Hemoglobin complex (Hp-Hb) shows considerable antioxidant property. In this study, antioxidant activity of Hp (2-2)-Hb complex on hydrogen peroxide has been studied and analyzed in the absence and presence of two beta-lactam antibiotics *in-vitro.* For this purpose, non-Michaelis behavior of peroxidase activity of Hp (2-2)-Hb complex was analyzed using Eadie-Hofstee, Clearance and Hill plots, in the absence and presence of pharmaceutical dose of ampicillin and coamoxiclav. The results have shown that peroxidase activity of Hp (2-2)-Hb complex is modulated via homotropic effect of hydrogen peroxide as an allostric substrate. On the other hand antioxidant property of Hp (2-2)-Hb complex increased via heterotropic effect of both antibiotics on the peroxidase activity of the complex. Both drugs also have mild effect on quality of homotropic property of the peroxidase activity of Hp (2-2)-Hb complex. Therefore, it can be concluded from our study that both beta-lactam antibiotics can increase peroxidase activity of Hp (2-2)-Hb complex via heterotropic effect. Thus, the two antibiotics (especially ampicillin) may help those individuals with Hp (2-2) phenotype to improve the Hp-Hb complex efficiency of removing hydrogen peroxide from serum under oxidative stress. This can be important in the individuals with phenotype Hp 2-2 who have less antioxidant activity relative to other phenotypes and are susceptible to cardiovascular disorders, as has been reported by other researchers.

## Introduction

During physiological and pathological hemolysis, hemoglobin (Hb) is released in plasma from red blood cells or their precursors (1). In healthy individuals physiological hemolysis accounts for ~10% of red blood cell destruction in the circulation, known as intravascular hemolysis (2). Moreover, the free-Hb may also increase by pathological hemolysis during some diseases such as hepatitis (3). The free-Hb represents a highly toxic substance, unless rapidly cleared from the circulation. This toxicity arises from the heme-iron, which can react with endogenous hydrogen peroxide to produce free radicals that may cause severe oxidative tissue damage and consequent disruption of cell function (4-6). On the other hand, iron content may decrease in the body because of glumerular filtration of the relatively small free-Hb molecules (7). 

Haptoglobin (Hp) is a plasma glycoprotein produced and released mostly by hepatocytes into the circulation. Haptoglobin irreversibly forms a soluble complex with free-Hb via non-covalent bonds. This binding is one of the strongest non-covalent interactions reported in biology (8-10). The formation of Hp-Hb complex has two main functions during hemolysis. As evidenced by studies in Hp knockout mice, it protects tissuses against heme-mediated oxidative damages, in particular in the kidneys (11, 12). The other is reduction of glomerular filtration of the free-Hb molecules after Hp-Hb complex formation (7). Eventually the Hp-Hb complex can be internalized by macrophages that express receptor for the complex (13) in order to recycling iron. 

In various diseases such as infectious diseases, oxidative stress occurs secondary to the initial disease but plays an important role in immune or vascular complications (14). Interestingly, the relative antioxidant effect of some *β*-lactam antibiotics such as ampicillin on oxygen-reactive species (ROS) has been reported and a possible therapeutic role for *β*-lactam agents in protecting host tissues from oxidative damage has been proposed (15). With regard to the extensive and strong antioxidant activity of Hp-Hb complex mentioned earlier, this complex is considered as an active part of body antioxidant defense system (16-18). Moreover, since the complex level in serum rises considerably after various diseases such as bacterial infection (19-21), it has been used as a biomarker in clinical diagnosis of those diseases (22, 23). Hp level in serum has also been subject of many researches in the presence and absence of antibiotics (24-27) which used as treatment of bacterial infections. But there has been a lack of information about antioxidant activity of Hp-Hb complex in the presence of antibiotics, especially *β*-lactam antibiotics which are related to host oxidative stress. 

As it has been mentioned before, heme-iron of free-Hb can react with endogenous hydrogen peroxide and produce free radicals (4), while Hp-Hb complexes have an ability to remove these hydrogen peroxide molecules by peroxidase activity (28). In this study, for the first time, kinetic analysis of peroxidase activity of Hp (2-2)-Hb complex has been performed in oxidative status induced by different concentrations of hydrogen peroxide. Moreover, effect of two *β*-lactam antibiotics (ampicillin and coamoxiclav) on the kinetics has been investigated as third parameter which is effective on oxidative condition (15). The data indicated that both antibiotics increased peroxidase activity of Hp (2-2)-Hb complex. 

## Experimental

*Materials*

*Hydrogen peroxide*

A solution of hydrogen peroxide, 0.5M was prepared in phosphate buffer 50 mM and pH 7.5. Peroxide solution is discarded after 30 min and if necessary a fresh dilution is prepared (29). 

*Guaiacol reagent*

A buffered solution of guaiacol, 0.03 M, is prepared as follows: 1.86 g of guaiacol and 50 mL of acetic acid are added to 400 mL of water. The volume is made up to 500 mL with water. The guaiacol reagent is stable for several weeks when stored in the cold (29).

*Met-hemoglobin solution*

Hemoglobin solution was prepared at a concentration of 10 mg/mL in phosphate buffer 50 mM and pH 7.5. An equal volume of 0.4 mM potassium ferricyanide is added to convert completely the oxy-hemoglobin to met-hemoglobin. The met-hemoglobin solution is then carefully diluted using the same buffer to 0.03 mM (29).

*Haptoglobin 2-2 solution*

A solution of Hp 2-2 (0.03 mM) was prepared in phosphate buffer 50 mM, pH 7.5 and the concentration of the solution was determined using reported extinction coefficient of haptoglobin 2-2 (ε mM^-1^ 58.65). The molar amount of Hp2-2 was based on its monomer properties because each Hp2-2 monomer is thought to be capable of binding a single hemoglobin molecule (30, 31).

*Preparation of Hp (2-2)-Hb complex*

Hp (2-2)-Hb complexes were formed by mixing 50 mL of each above mentioned solution of Hp2-2 and Hb, incubating for 30 min at room temperature with gentle agitation to ensure that all Hp molecules was saturated with Hb. 

*Enzymatic activity assay*

Peroxidase activity of Hp (2-2)-Hb complex was assayed by following increase of absorption of produced tetraguaiacol as the second substrate of Hb-Hp complex at 470 nm and 37^˚^C by UV-Vis spectrophotometer (CICEL Model 9000) (32). The assay mixture contained Hp (2-2)-Hb complex 64 nM, 0.03 M guaiacol and different concentration of H_2_O_2_ in phosphate buffer 50 mM, pH 7.5 in 1 mL final volume. All components except for Hp-Hb complex were added directly to a quartz cuvette and mixed by inverting 6 times. Five micro liters Hp-Hb solution was then added to the mixture, and the cuvette was inverted three times to mix the ingredients. The zero time point for following A_470 _was designated as the time at which Hb-Hp complex was added to the solution. For each concentration of hydrogen peroxide, the reaction was performed at least 3 separate times. To investigate the effect of antibiotic assay mixtures contained 14 µg/mL ampicillin and coamoxiclav (20 g/mL amoxicillin, and 4 g/mL clavolonic acid) which corresponded to their maximum concentrations in the plasma after injection.

*Determination of enzymatic parameters*

Maximum initial velocities (V_max_) were obtained graphically using appropriate saturation curves. The amount of S_50 _(a concentration of H_2_O_2_ in which velocity of enzymatic activity is half of V_max_) were obtained from the H_2_O_2 _concentration related to cross point of Hill plot to X-axis. The amount of (CL_max_) (maximum clearance) and S_max _(a concentration of H_2_O_2_ in which CL_max _occurs) were graphically obtained using clearance plots.

*Calculation of V-cal*

Calculated initial velocity (V-cal) was obtained using Hill equation:

$V=\frac{V_{\max}\times S^{m}}{S_{50}^{m}+S^{m}}$

Where V_max_ is maximum initial velocity, S_50_ is the substrate concentration corresponding to half of V_max_, m is Hill coefficient (slope of Hill plot) and S is various substrate concentrations (33).

## Results and discussion

Although several physiological roles have been referred to Hp, the primary function of Hp still appears to be binding to free-Hb and its antioxidant activity, therefore protecting from heme-catalyzed oxidative stress and facilitating Hb-uptake by macrophages. Haptoglobin as an acute phase plasma glycoprotein (22) has two allelic sites producing three phenotypes: Hp1-1, Hp2-2 and Hp1-2. Individuals with phenotype1-1 and 2-2 have maximum and minimum antioxidant activity, respectively. Consequently, patients with Hp2-2 phenotype who suffer from chronic diseases which are related to oxidative stress are more susceptible to cardiovascular disorders (34). According to some studies less antioxidant activity of Hp2-2 can be compensated with antioxidant effects of some additives such as glutathione peroxidase (35) and vitamin E (36). 

Antioxidant activity of Hp-Hb complex is an important factor, not only in chronic diseases, but also in some acute infections with considerable increase in serum level of Hp (19-23). With regard to the wide-spread use of antibiotics for treatment of various infectious diseases, their effects as an additional factor on antioxidant property of Hp-Hb complex can be of interest (24-27), especially if it is considered that, importantly, antibiotic susceptibility of bacteria is also related to oxidative status (37-39). Here we have studied on the effect of the presence of two *β*-lactam antibiotics to investigate probable increasing in peroxidase activity of Hp (2-2)-Hb. 

**Figure 1:**
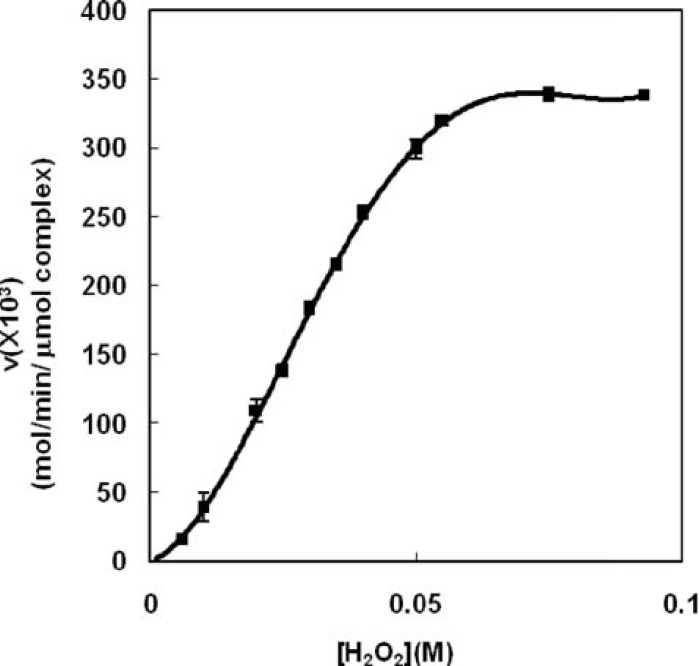
Saturation curve for antioxidant activity of Hp (2-2)-Hb using hydrogen peroxide as substrate in phosphate buffer 50 mM, pH 7.5 at 37°C. Each enzymatic assay was done using 0.03 M guaiacol as second substrate and following of A_470_. Sigmoid shape of the curvature shows non-Michaelis behavior

Because of the lack of kinetic studies on peroxidase activity of Haptoglobin-Hemoglobin complex, the present study has investigated kinetic properties of the complex enzymatic activity for the first time. The saturation curve of Hp (2-2)-Hb complex shows a sigmoid shape (Figure 1) indicating the activity of Hp (2-2)-Hb complex could not be analyzed using Michaelis-Menten model and requires the adoption of an enzyme model with multiple sites (33) showing cooperative binding for the substrate (H_2_O_2_). 

**Figure 2:**
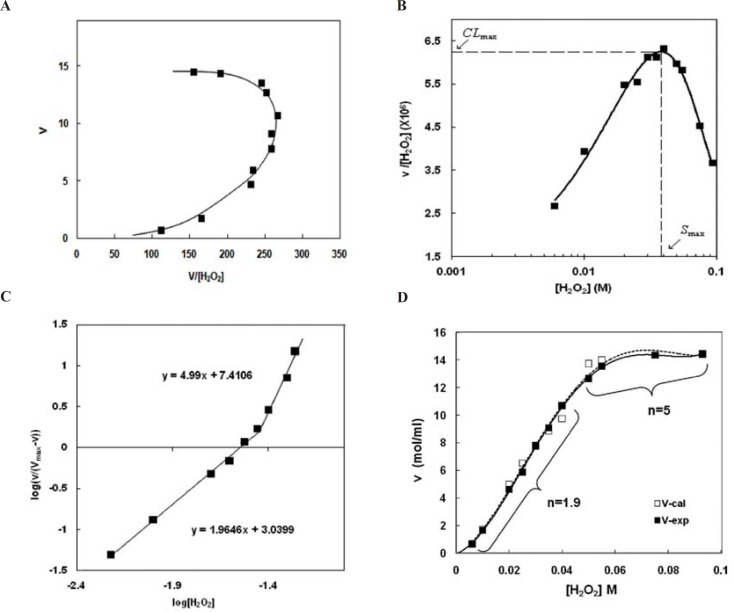
Non-Michaelis analysis of peroxidase activity of Hp (2-2)-Hb complex. A) Eadie-Hofstee plot. The nonlinear shape and the curvature toward right show non-Michaelis homotropic allostric effect. B) Clearance plot. As it is observed the upward curvature confirms homotropic property. Maximum clearance (CL_max_) and S_max_ have been determined graphically as shown. C) Hill plot. It is seen the points of this graph lay on at least two consecutive linear parts. The slope of each line (*m*) is more than unit (*m*>1). This observation not only confirms positive cooperativity and homotropic effect but also demonstrates the behavior is changed with H_2_O_2_ concentrations so that the two sequential homotropic behaviors lead to increased activity. D) comparison between experimental and calculated saturation curve. Experimental initial velocities (-) are directly from figure 1. Calculated initial velocities (…..) have been calculated using Hill coefficients (Fig 2,C) and Hill equation as explained in Materials and Methods

Eadie-Hofstee plot (Figure 2.A) confirms positive cooperativity deduced from the right-side curvature of the plot. This indicates that H_2_O_2 _not only is a substrate of the peroxidase activity, but also plays as an activator at higher concentrations of H_2_O_2_ Figure 2.B shows that there is a well defined maximum for the clearance of the substrate, CL_max_. It is worthy to note that CL_max_ is determined under *in-vitro* condition but it is appropriate parameter for investigation of the activity under *in-vivo* condition. The concentration of hydrogen peroxide that causes maximum clearance (CL_max_) is named S_max_. Both of the parameters were obtained from Clearance plot (Figure 2.B). S_max_ was observed in relatively low hydrogen peroxide concentrations, because of the maximum complex autoactivation. Indeed, CL_max_ provides an estimate of the highest clearance attained as substrate concentration increases before saturation of the enzyme sites. 

Thus if the assumption is made that *in-vivo *activation occurs via endogenous activators, then CL_max_ may be an appropriate parameter for describing the salient feature of the subsystem that can be used for predictive purposes (33). 

Hill plot(Figure 2.C) not only confirms positive allostric effect (Hill coefficients are more than 1, *m*>1), but also shows that there are two sequential positive allostric effects (*m*_1_=1.9, m_2_=5) with increasing H_2_O_2_ concentration (Table 1). 

**Table 1:** Enzymatic and allosteric parameters of antioxidant activity of Hp (2-2)-Hb complex in the presence of ampicillin and coamoxiclav. The values have been directly obtained from saturation curve (Figures 1 and 3) Clearance plots (Figures 2.B, 4.B) and Hill plots (Figures 2.C, 4.C, 4.D) as it has been mentioned in “Materials and Methods”.

***m***_2_	***m***_1_	**S**_50 _ **(M)**	**S**_max _ **(M)**	**CL**_max _ **(**_10_^6^ **)**	**Antibiotic**
5.0	1.9	0.03	0.04	6.3	-
2.8	1.9	0.03	0.04	7.3	ampicillin
7.8	1.9	0.03	0.05	6.8	coamoxiclav

Calculated initial velocities (V-cal) obtained from Hill equation using the Hill coefficients (see Material and Method) is in good coincidence with experimental initial velocity (V-exp) (Figure 2.D).

**Figure 3:**
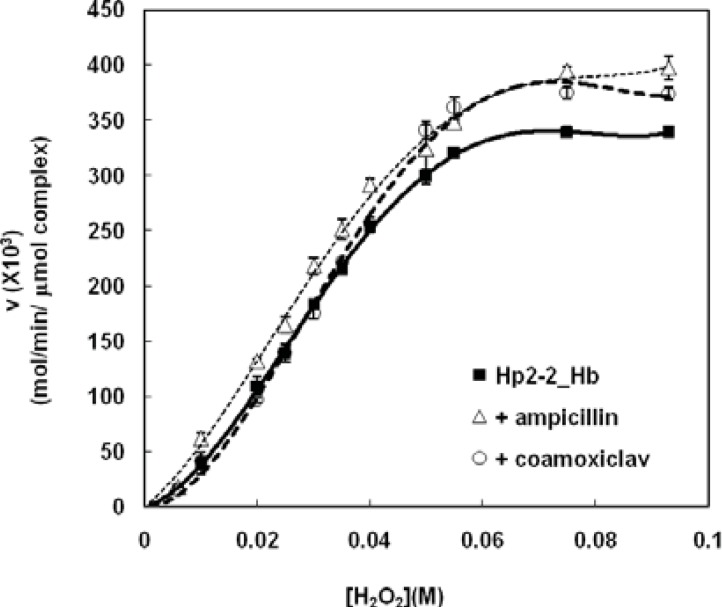
Saturation curves for peroxidase activity of Hp (2-2)-Hb complex using hydrogen peroxide as substrate in phosphate buffer 50 Mm , PH 7.5 at 37°C in the presence of therapeutic concentration ampicillin and coamoxiclav . Saturation curve of Figure 1 has been incorporated here, for comparision

As it is shown in Figure 3; both ampicillin and coamoxiclav have activating effect on Hp (2-2)-Hb peroxidase activity (heterotropic effect or heteroactivation), but they have not changed the sigmoid shape of saturation curve (homotropic effect or autoactivation). It seems that both drugs induce a new conformation to the complex which is more active. In other word, autoactivation and heteroactivation have occurred simultaneously.

**Figure 4:**
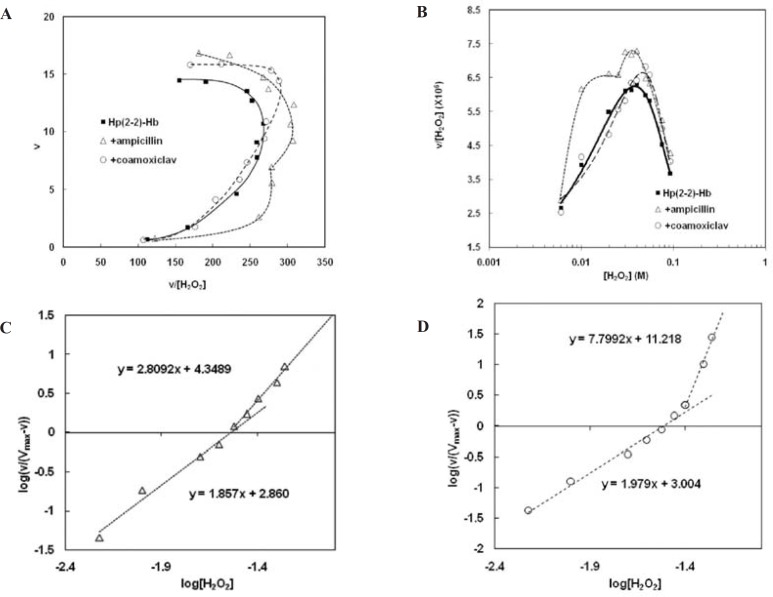
Non-Michaelis analysis of peroxidase activity of Hp (2-2)-Hb complex. A) Eadie-Hofstee plot. The nonlinear shape and the curvature toward right show non-Michaelis homotropic allostric effect. Eadie-Hofstee plot in the absence of drugs (Figure 2.A) has been incorporated here, for comparisoan.B) clearance plot. As it is observed the upward curcature confirms homotropic property. Maximum clearance (CL_max_) and S_max_ have been determined graphically as shown. Clearance plot in the absence of drugs (Figure 2.B) has been incorporated here, for comparison. C) Hill plot in the presence of ampicillin. It is seen the points of this graph lay on at least two consecutive linear parts. The slope of each line (*m*) is more than unit (*m*>1). this observation not only confirms positive cooperativity and homotropic effect but also demonstrates the behavior is changed with H_2_O_2_ concentrations so that the two sequential homotropic behaviors lead to increased activity. D) Hill plot in the presence of coamoxiclav. The same effect can be observed here Moreover the slope of second line in the presence of coamoxiclav, is more than of ampicillin. the obtained values of CL_max_, S_max _and Hill coefficients have been summarized in Table1.

Analysis of the Hp (2-2)-Hb complexsaturation curves in the presence of the two antibiotics (Figure 4), has shown that Hp (2-2)-Hb complex maintains the homotropic behavior of its peroxidase activity. Moreover the drugs have not changed the first Hill coefficient, while the second Hill coefficient has been decreased in the presence of ampicillin and increased in the presence of coamoxiclav (Figure 4.C and 4.D, respectively). These results have been summarized in Table1. Comparison of the amounts of CL_max _shows that both antibiotics increase CL_max _of complex to remove H_2_O_2_ (Figure 4.B, Table1). This effect in the presence of ampicillin is two times more than of coamoxiclav (about 16% increasing in peroxidase activity in the presence of ampicillin and 8% in the presence of coamoxiclav). S_max _has been shifted to relatively higher concentrations of H_2_O_2 _in thepresence of coamoxiclav, while ampicillin has not considerable effect on S_max _(Figure 4.B, Table1). This indicate that maximum efficiency of Hp (2-2)-Hb complex for the clearance of H_2_O_2_ has been shifted to the higher concentrationsof H_2_O_2_. S_50 _has not been changed in the presence of the two drugs (Table1). 

In conclusion, ampicillin and coamoxiclav can act as activators for peroxidase activity of Hp (2-2)-Hb complex via heterotropic effect. It will be meaningful if we remind that the two antibiotics are usually used under pathologic oxidative status caused by bacterial infections (19-21), and *β*-lactam antibiotics such as ampicillin have been shown to affect on their host oxidative status (15). This can be important in the individuals with phenotype Hp2-2 who have less antioxidant activity relative to other phenotypes and are susceptible to cardiovascular disorders (34). Therefore our *in-vitro* studies show that the two antibiotics (especially ampicillin) may help those individuals with Hp (2-2) phenotype to improve the Hp-Hb complex efficiency of removing hydrogen peroxide from serum under oxidative stress. This study needs to be continued by *in-vivo* experiments to confirm the conclusion under physiologic condition.
